# Inhibition of breast cancer metastasis to the lungs with UBS109

**DOI:** 10.18632/oncotarget.26302

**Published:** 2018-11-16

**Authors:** Mamoru Shoji, Wei Ping Qian, Ganji Purnachandra Nagaraju, Daniel J. Brat, Danielle Pessolano, Rick Luzietti, Spandan Chennamadhavuni, Masayoshi Yamaguchi, Lily Yang, Dennis C. Liotta

**Affiliations:** ^1^ Department of Hematology and Medical Oncology, Winship Cancer Institute, Emory University, Atlanta, GA 30322, USA; ^2^ Department of Surgery, Emory University, Atlanta, GA 30322, USA; ^3^ Department of Pathology, Northwestern University Feinberg School of Medicine and Northwestern Memorial Healthcare, Chicago, IL 60611, USA; ^4^ Agilux Laboratories, Inc./Charles River Laboratories, Inc., Worcester, MA 01608, USA; ^5^ Emory Institute for Drug Development, Emory University, Atlanta, GA 30322, USA; ^6^ Department of Chemistry, Emory University, Atlanta, GA 30322, USA

**Keywords:** MACs, UBS109, breast cancer, lung metastases, bone metastasis

## Abstract

Synthetic monocarbonyl analogs of curcumin (MACs) are cytotoxic against several cancers including head and neck cancer, pancreatic cancer, colon cancer, and breast cancer. Mechanisms of action include depolarization of the mitochondrial membrane potential and inhibition of NF-κB, leading to apoptosis. We previously demonstrated that UBS109 (MAC), has preventive effects on bone loss induced by breast cancer cell lines. We determined whether UBS109 could inhibit and prevent lung metastasis, since lung metastasis of breast cancer is a major problem in addition to bone metastasis. A breast cancer lung metastasis (colonization) model was created by injection of breast cancer cells MDA-MB-231 into the tail vein of athymic nude mice, nu/nu. Animals were treated with vehicle or UBS109 at 5 or 15 mg/kg body weight by intraperitoneal injection once daily 5 days a week for 5 weeks. UBS109 at 15 mg/kg significantly inhibited lung metastasis/colonization as demonstrated by reduced lung weight consisting of tumor nodules. The body weight of animals treated with UBS109 15 mg/kg remained the same as in the other groups. UBS109 killed completely (100%) MDA-MB-231 breast cancer cells at 1.25 μM in a cytotoxicity assay *in vitro*. UBS109 15 mg/kg i.p. showed a maximal blood concentration (C_max_) of 432 ± 387 ng/mL at 15 min post injection. This is approximately 1.5 ng/ml in the blood of mice and equals 1.5 μM of UBS109. These *in vitro* and *in vivo* results are consistent with each other.

## INTRODUCTION

**Figure 1:**
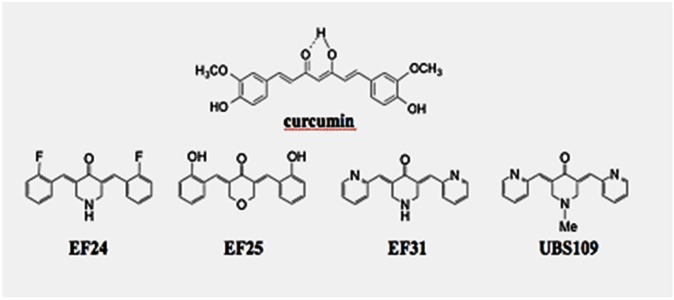
Chemical structures of the monocarbonyl analogs of synthetic curcumin (MACs), EF24, EF31, and UBS109 Molecular weights of curcumin, EF24, EF31, and UBS109 are 368.38, 311.11, 277.12, and 291.35 g/mol, respectively. Cytotoxic activity and solubility of the MACs are described [11].

Curcumin (diferuloylmethane) is a polyphenol derived from the plant *Curcuma longa*, commonly called turmeric. The compound has been reported to have anti-oxidative, anti-tumor promoting, anti-thrombotic and anti-inflammatory properties. The pleiotropic effects of curcumin are attributed at least partially to inhibition of the transcription factors, nuclear factor-κB (NF-κB), AP-1 and Egr-1 and their downstream signals [1–4]. We prepared over 100 monocarbonyl analogs of curcumin (MACs) in order to preserve the remarkable diversity of curcumin's biological actions and improve its weak potency, low solubility and poor bioavailability, and screened for anticancer and anti-inflammatory activities. The National Cancer Institute (NCI) screened 13 MACs against the NCI-60 cancer cell line panel. One of the MACs, EF24, exhibited much stronger anticancer activity than cisplatin with less toxicity [5]. The mean growth inhibitory concentration GI_50_ of EF24, curcumin and cisplatin were measured to be 0.7 μM, 7.3 μM, and 9.5 μM, respectively [5–7]. Hence, EF24 showed much stronger anti-cancer activity than cisplatin. The NCI subcontracted the Mayo Clinic Department of Pharmacology to perform a pharmacokinetic study of EF24 [8]. Since then, we have developed much more active analogs (Figure 1). Furthermore, MACs including EF24, EF31 and UBS109 inhibit NF-κB by suppressing IκB kinase-α and -β (IKK-α and -β) [7, 9–16]. UBS109 is the most water soluble among the MACs and shows excellent activity against xenografts of head and neck squamous cell carcinoma, pancreatic cancer, colon cancer, and breast cancer [11, 15, 16]. UBS109 significantly inhibits the translocation of NF-κB in many malignant cell lines compared to other curcumin analogs [7, 9–16]. Furthermore, it decreases the metastatic properties of tumors, i.e. angiogenesis, invasion and migration, by decreasing their associated signalling pathways.

The bones, lungs, liver, and brain are frequent sites of breast cancer metastasis [17]. Breast cancer bone metastasis occurs in 70–80 % of patients with advanced breast cancer [18], leading to severe pathological bone fractures, pain, hypercalcemia, and spinal cord and nerve-compression syndromes [19, 20], which are a common cause of morbidity and mortality. Tumor invasion into bone tissues is associated with osteoclast and osteoblast recruitment, resulting in the liberation of growth factors from the bone matrix, which can feed back to enhance tumor growth resulting in the vicious cycle of bone metastasis [17–22].

Recently, we demonstrated that the UBS109 has preventive effects on bone loss induced by breast cancer cell lines. This bone loss was prevented by p.o. and i.p. administration of UBS109 *in vivo*. UBS109 has suppressive effects on osteoclastogenesis by antagonizing RANKL-induced NF-κB activation and potent stimulatory effects on osteoblastogenesis and mineralization through activation of Smad signaling [9, 10, 12].

Since breast cancer lung metastasis is a major problem in addition to bone metastasis, we studied the efficacy of UBS109 on lung metastasis of breast cancer MDA-MB-231 cells. We injected breast cancer cells into the tail vein of athymic nude mice to generate a lung metastasis (colonization) model for the study of UBS109 activity [23]. UBS109 at 15 mg/kg i.p. administration inhibited lung metastases.

## RESULTS

### Selection of UBS109 dose and formulation

Chemical structures of curcumin, EF24, EF31, and UBS109 are shown in Figure 1. Cytotoxic activities against MDA-MB-231 cells were shown previously [11]. We have previously determined the maximally tolerated dose (MTD) of UBS109 to be between 20 – 25 mg/kg body weight [9]. In our previous study, mice received 0, 15, 20 and 25 mg/kg body weight/0.1 ml, i.p. twice a week (n = 4/dose). UBS109 was dissolved in 1 mL of 100% ethanol and diluted to the desired concentrations with PBS. It appeared that mice were more susceptible to ethanol as a diluent than DMSO. Thus, we diluted UBS109 in DMSO as described by Zhu et al [11]. Therefore, in this study we selected the maximal dose of UBS109 of 15 mg/kg, i.p. since we planned to administer UBS109 i.p. once daily for 5 days a week for 5 weeks.

### Efficacy of UBS109 against lung metastasis of breast cancer MDA-MB-231 xenografts

**Figure 2:**
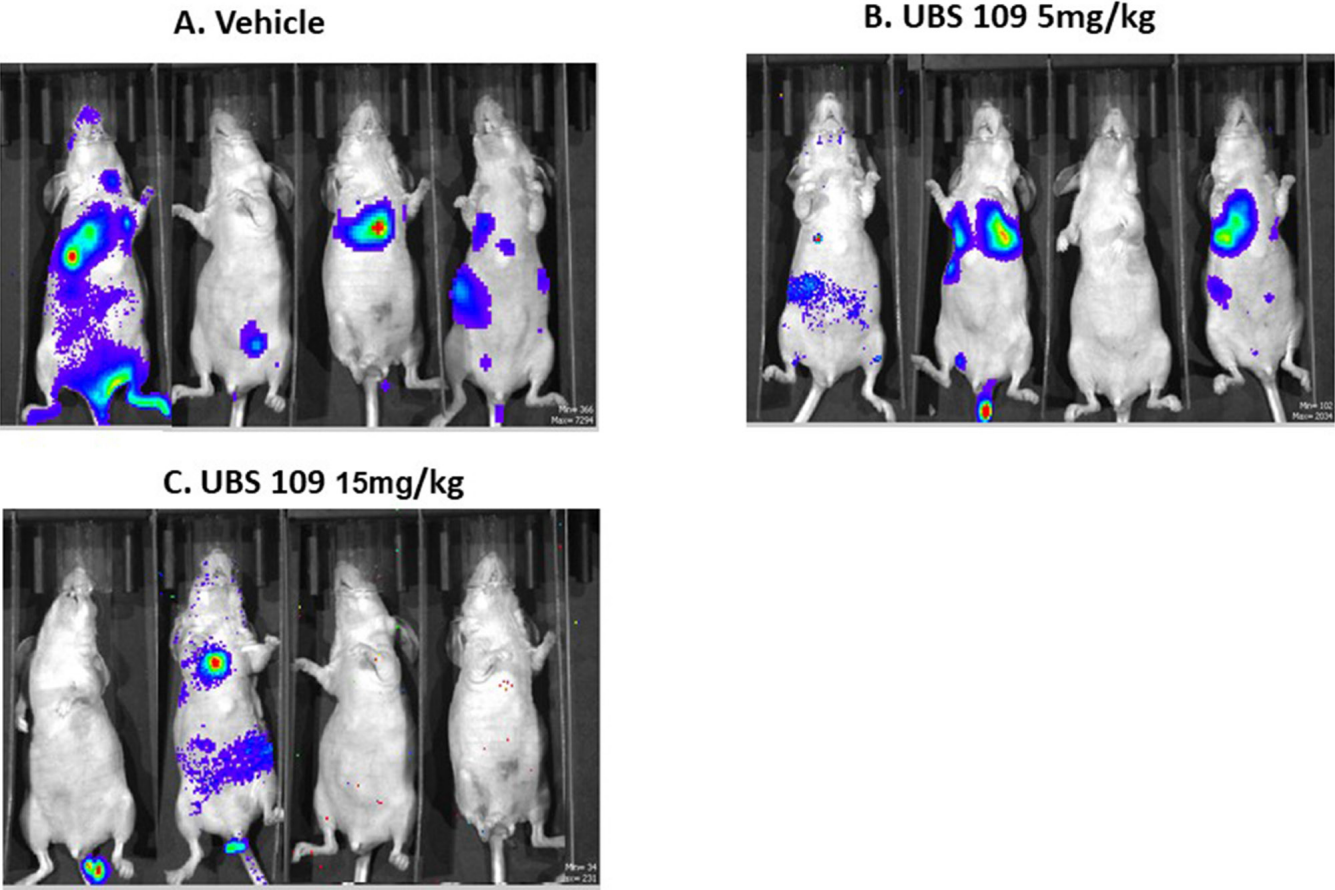
The luciferase activity in breast cancer cells The luciferase activity of breast cancer cells and their distribution after injection of cells into the tail vein of mice. Mice were treated with intraperitoneal injection of vehicle **(A)** or UBS109 at 5 or 15 mg/kg/body weight **(B)** or **(C)** once daily for 5 days per a week (from Monday through Friday) for approximately 5 weeks (n = 4/treatment regimen).

**Figure 3:**
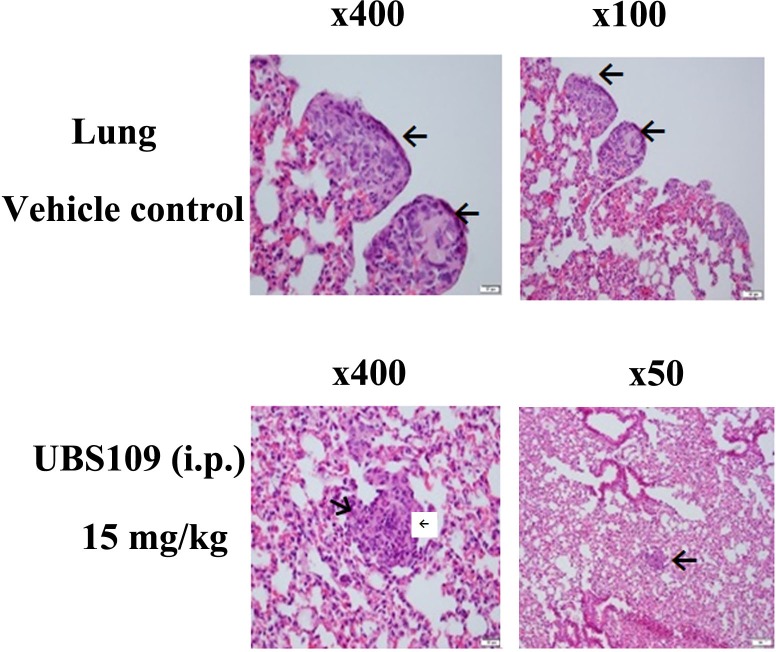
Hematoxylin and eosin stains of the breast tumors in the lungs Hematoxylin and eosin stains of the breast tumors in the lungs from the vehicle- or UBS109 (15 mg/kg, i.p.)-treated mice. Arrows indicate the tumors. Number indicates the total magnification of the image through the microscope.

The effect of UBS109 administration on lung metastasis was examined in mice bearing MDA-MB-231 xenografts in the lungs. Animals were administered therapeutic regimens of UBS109 by i.p. once/day 5 days/week (M-F) for 5 weeks, in the following groups: control vehicle, UBS109 at 5 mg/kg in vehicle, and UBS109 15 mg/kg in vehicle (n = 4). Figure 2 shows the luciferase activity of breast cancer cells and their distribution after injection of cells into the tail vein of mice. The cancer cells were mainly found in the lungs and some other sites. Mice treated with UBS109 at 15 mg/kg exhibited much less colonization/metastasis in the lungs. Treatment with UBS109 15 mg/kg appears the most efficacious in inhibiting tumors (lung colonies) (Figures 2 and 3). The tumor deposition in the lungs measured by weight was significantly reduced by treatment with UBS109 at 15 mg/kg body weight, i.p., but not 5 mg/kg (Table 1). Lung weight was used to assess the breast cancer colony load present in the lungs of each mouse, since it is difficult to accurately count all colonies outside and inside in each lung. Figure 3 shows hematoxylin-eosin (H&E) stains of the lungs of mice treated with vehicle or UBS109 at 15 mg/kg. The UBS109-treated lung showed smaller and fewer colonies, whereas the vehicle-treated lung showed larger and more abundant colonies.

**Table 1:** Effect of UBS109 on the lung weight (breast tumor nodules)

Treatment	Lung weight (mg)
Vehicle control (n=4), ip	296.6 ± 18.8
UBS109 5 mg/kg (n=4), ip	285.9 ±11.2
UBS109 15 mg/kg (n=4), ip	226.6 ± 19.8^*^
	*p<0.05*

^*^p<0.05, significantly less from control, i.e., less metastatic breast tumor nodules grow in the lungs; ± indicates S.D.

**Figure 4:**
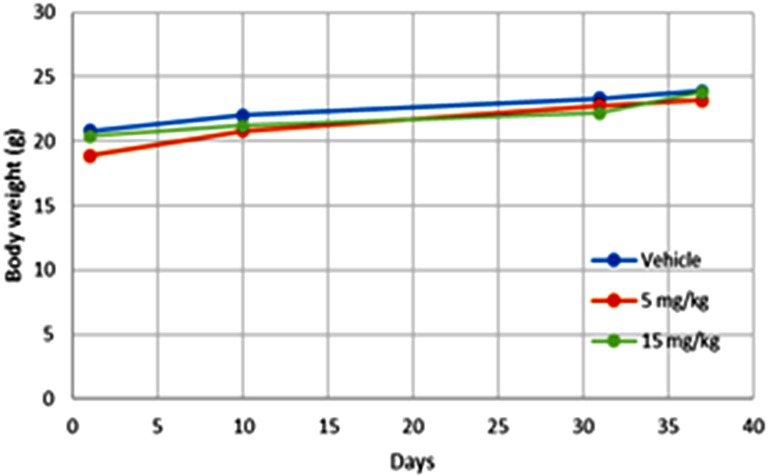
Body weight (gram) of the mice Body weight (gram) of the mice during treatment with UBS109 (at 5 or 15 mg/kg body weight, i.p.) or vehicle.

Treated animals did not lose weight during treatment over 5 weeks, but rather gained weight similar to controls (Figure 4). This suggests that UBS109 treatment was not toxic at 5 or 15 mg/kg body weight i.p. once daily 5 days a week for 5 weeks. These findings suggest that UBS109 may be a promising agent for the prevention and inhibition of metastasis for patients with breast cancer.

### Preclinical pharmacokinetic (PK) studies of UBS109 15 mg/kg by i.p

**Figure 5:**
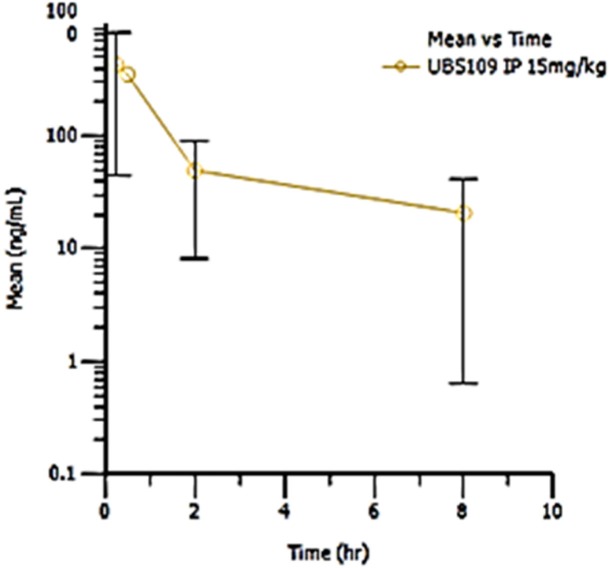
Pharmacokinetic study of UBS109 Mean UBS109 plasma concentration (ng/mL) at 0.5, 2 and 8 hours after a single i.p. injection of UBS109 at 15 mg/kg body weight. This pharmacokinetic study was performed by the Agilux Lboratories, Worcester, MS 01608, USA (incorporated now with the Charles River Laboratories).

Pharmacokinetic analysis of the plasma concentration time curve for each animal was conducted using noncompartmental analysis with WinNonlin® Version 6.3.0 (Figure 5). UBS109 15 mg/kg i.p. showed mean and maximal blood concentration (C_max_) at 15 min post injection (0.25 hr) of 432 ± 387 ng/mL (C_max_). This is approximately 1.5 ng/ml in the blood of mice and, given the molecular weight of UBS109 of 291.4, yields a blood concentration of approximately 1.5 μM. We previously demonstrated 100% cell killing of MDA-MB-231 cells *in vitro* by UBS109 at 1.25 μM [11]. Thus the *in vitro* and *in vivo* data are consistent, and suggest that treatment with UBS109 15 mg/kg i.p. daily 5 days a week for 5 weeks may be efficacious.

## DISCUSSION

We used a lung metastasis (colonization) model to quantify the effect of UBS109 by measuring lung weights [23]. The majority of tumor cells are deposited in the lungs but a few cells are deposited in the liver and other areas through the vein on the way to the lungs (Figure 2). Although natural metastasis occurs during the growth of a primary tumor and is manifest after removal of the primary tumor, it is difficult to quantify the efficacy of UBS109 treatment in this setting since metastases will spread to different parts of the body in each animal. We have demonstrated promising efficacy of UBS109 in the treatment of metastasis and will now be able to use a model similar to the natural metastasis process to determine the effect of UBS109.

In addition to breast cancer, MACs are effective against most other cancers as demonstrated by the NCI-60 cancer cell line studies [5]. Cisplatin has been used for the treatment of several human cancers including bladder, head and neck, lung, ovarian, and testicular cancers [24]. However, the NCI demonstrated that MAC EF24 has much stronger anticancer activity than cisplatin against all NCI-60 cell lines [5]. UBS109 is the most active among MACs including EF24 and EF31, and exhibited very strong anti-cancer activity against pancreatic cancer, colon cancer, and head and neck cancer [11, 15, 16]. We tested the cytotoxic activities of five anticancer drugs including an Akt inhibitor (MK2206), EF24, gemcitabine (the current chemotherapeutic drug for pancreatic cancer), HSP90 inhibitor (STA9090), p38 MAPK inhibitor, and UBS109 against four different human pancreatic cancer cells MiaPaca-2 (no tissue factor (TF)-expressed), ASPC-1 (full length TF expressed), PT45P1 (full length TF expressed) and PT45P1/asTF+ (alternatively spliced TF over-expressed) in collaboration with Dr. Vladimir Y Bogdanov, University of Cincinnati. The ranking of the cytotoxic activities was UBS109> EF24> HSP90 inhibitor> gemcitabine> Akt inhibitor> p38 MAPK inhibitor. UBS109 inhibited all four pancreatic cancer cell lines 100% at 0.25 μM, whereas gemcitabine only killed the cells maximally above 50% up to 20 μM used in the test [16]. Therefore, UBS109 may be efficacious against pancreatic cancer, colorectal cancer, and metastatic breast cancer and can be applied to the treatment of many other cancer types among the NCI-60 cancer cell line panel.

## MATERIALS AND METHODS

Dulbecco's Modification of Eagle's Medium (DMEM) and antibiotics (penicillin 10,000 units/mL and streptomycin 10,000 μg/mL) were purchased from Invitrogen (Carlsbad, CA, USA). Fetal bovine serum (FBS) was obtained from Hyclone. UBS109 (3,5-bis(2-pyridinylmethylidene)-1-methyl-4-piperidone) was prepared at Emory University; its chemical structure was shown previously (Figure 1) [9]. UBS109 was dissolved in 100% ethanol to use in cell culture experiments. All other reagents were purchased from Sigma-Aldrich (St. Louis, MO, USA), unless otherwise specified.

### Synthesis of UBS109

We described the synthesis of UBS109 in detail in our previous studies [9, 11].

### Breast cancer cells

We used the triple-negative MDA-MB-231 cell line containing the luciferase gene (MDA-MB-231-luc-D3H2LN) purchased from the Caliper Life Sciences (Hopkinton, MS, USA). The cells were cultured in DMEM supplemented with 10% fetal bovine serum (FBS) (Gibco, 26140-079), penicillin (100 units/mL), streptomycin (100 μg/mL) and 2 mM L-glutamine. Cells were incubated at 37°C in 5% CO_2_/95% air in a humid atmosphere.

### Animals

Female athymic nude mice (nu/nu, Harlan Sprague-Dawley strain; 5-6 weeks old) were housed in a pathogen-free facility. All procedures and protocols were approved by the Institutional Animal Care and Use Committee at Emory University. Mice were randomized into three groups: normal group (no tumor cells injected), lung metastasis model group (vehicle control), and two UBS109-treated groups with different dosages, 5 and 15 mg/kg body weight, 4 mice in each group.

### UBS109 treatment of breast cancer cells for lung metastasis

Athymic nude mice were housed in a pathogen-free facility and were intravenously injected into the tail vein with breast cancer MDA-MB-231 bone metastatic cells (10^6^ cells/mouse) to result in lung metastasis (colonization). One week following injection, mice were randomized to receive either vehicle (0.5% carboxymethyl cellulose sodium [CMC] with 10% DMSO in sterile water) or drug (UBS109). The agents (vehicle, UBS109 at 5 or 15 mg/kg body weight) were administered intraperitoneally (i.p.) daily 5 days per week for 5 weeks (n = 4 mice/regimen). CMC was dissolved in distilled water and sterilized by autoclaving. Due to chemical instability, a stock solution of the drugs was made fresh each time, by weighing out 100 mg UBS109 and dissolving it in 1 mL of 100% DMSO by briefly heating before making the appropriate dilution. After UBS109 administration for 5 weeks, animals were sacrificed. The lungs containing breast cancer tumors were weighed and fixed in 10% neutralized formalin for 48 h. Paraffin embedded blocks were stained with hematoxylin and eosin (H&E). Body weights were measured once a week.

### Pharmacokinetic study

A pharmacokinetic (PK) study was performed by Agilux Laboratories Inc./Charles River laboratories, Inc. (Worcester, MA, USA). Male CD-1 mice (Harlan Laboratories, Inc.) weighing 28 ± 1.6 grams were used for the PK study. Three mice were administered a single i.p. dose of UBS109 15 mg/kg in 10% DMSO and 90% PBS.

A blood sample (approximately 35–40 μL) was taken from each animal from the tail vein at 4 different time points after dosing: 0.25, 0.5, 2, and 8 h (n=3 mice/time point). Samples were collected into K2EDTA-coated Microvette tubes (Sarstedt) on wet ice (protected from light) and processed to plasma by centrifugation (3500 rpm at 5°C) within 1 hour of collection. All plasma samples were transferred into separate 96 well plates (matrix tubes) and stored at −80°C until analysis for the parent drug by Agilux Laboratories/Charles River laboratories, Inc. via LC/MS/MS.

The plasma samples (10 μL) were deproteinated with acetonitrile (80 μL) and analyzed by LC/MS/MS using an internal standard (carbutamide) spiking technique with an AB SCIEX 6500+ system equipped with a Waters Acuity BEH C18 UPLC column (2 × 50 mm, 1.7 μm) operating at 55°C with mobile phases A (95:5:0.1 H_2_O:CAN:FA) and B (50:50:0.1 CAN:MeOH:FA). The corresponding time courses were 1.40 and 2.10 min (%MPB 95; Flow 0.800 mL/min) followed by a 2.80 min Strong Needle Rinse (25:25:25:25:0.1 (v:v:v:v:v) MeOH:ACN:IPA:H_2_O:NH_4_OH). The verified research grade bioanalytical assay method to determine the plasma concentrations of UBS109 (RGA Level 1) employed an API 6500+ electrospray, positive ion MS/MS device. The mean, standard deviation, and %CV of plasma levels were determined using Microsoft^®^ Office Excel 2007. Mean concentration data was plotted against time to create plasma concentration versus time profiles at each dosage (Table 2 and Figure 5).

**Table 2:** Pharmacokinetic study of UBS109 administered by i.p

UBS109 IP dose (15mg/kg)						
Mice	T ½ (hr)	Tmax (hr)	Cmax (ng/mL)	AUClast (hr^*^ng/mL)	AUCinf (hr^*^ng/mL)	Extrapolated (%)
1	NR	0.25	398	813	NR	NR
2	1.97	0.25	623	116	124	6.15
3	NR	0.25	835	1061	NR	NR
Mean	1.97	0.25	618.67	664	124	6.15
SD	NA	0.00	218.53	490	NA	NA

Abbreviations: NR = Not reported due to insufficient characterization of the terminal phase of the plasma-concentration profile (Rsq < 0.9); NA = Not applicable; t½ = terminal half-life; AUC0-∞ = area under a concentration of analyte vs. time calculated using zero to infinity; AUC0-last = computed from time zero to the time of the last positive Y value; Cmax = the peak or maximum concentration; Tmax = the time of peak concentration; SD = standard deviation.

Pharmacokinetic analysis of the plasma concentration time curve for each animal was conducted using noncompartmental analysis with WinNonlin® Version 6.3.0. Areas under the curve (AUC) were estimated using the linear trapezoidal rule. The first order rate constant (Lambda Z) associated with the terminal (log-linear) elimination phase was estimated using linear regression of at least three nonzero concentrations versus time points in the late phase of the log concentration versus time profile. The half-life (t.) was calculated as ln(2)/Lambda Z. Nominal times were used in all the data analyses.

### Statistical analysis

Multiple comparisons were performed by one-way analysis of variance (ANOVA) with the Tukey-Kramer multiple comparisons post-hoc test for parametric data as indicated. *p*<0.05 was considered statistically significant.
